# Peptide Receptor Radionuclide Therapy During the COVID-19 Pandemic: Are There Any Concerns?

**DOI:** 10.2967/jnumed.120.249136

**Published:** 2020-08

**Authors:** Lisa Bodei, Emily Bergsland, Wouter W. de Herder, Diego Ferone, Rodney J. Hicks, Thomas A. Hope, Jolanta Kunikowska, Marianne Pavel, Diane Reidy-Lagunes, Jens Siveke, Jonathan Strosberg, Ulf Dittmer, Ken Herrmann

**Affiliations:** 1 Molecular Imaging and Therapy Service, Department of Radiology, Memorial Sloan Kettering Cancer Center, New York, New York; 2 Division of Hematology/Oncology, Department of Medicine, University of California San Francisco, San Francisco, California; 3 Erasmus MC and Erasmus MC Cancer Center, ENETS Center of Excellence Rotterdam, Rotterdam, The Netherlands; 4 Endocrinology Unit, IRCCS Ospedale Policlinico San Martino, and Department of Internal Medicine and Medical Specialties, University of Genova, Genova, Italy; 5 Sir Peter MacCallum Department of Oncology, University of Melbourne, Melbourne, Victoria, Australia; 6 Department of Radiology and Biomedical Imaging, University of California, San Francisco, San Francisco, California; 7 Nuclear Medicine Department, Medical University of Warsaw, Warsaw, Poland; 8 Department of Medicine 1, Endocrinology, Friedrich-Alexander University Erlangen-Nürnberg, Erlangen and Nürnberg, Germany; 9 Gastrointestinal Oncology Service, Department of Medicine, Memorial Sloan Kettering Cancer Center, New York, New York; 10 Institute for Developmental Cancer Therapeutics, West German Cancer Center, University Hospital Essen, Essen, Germany; 11 Department of Gastrointestinal Oncology, Moffitt Cancer Center, Tampa, Florida; 12 Institute for Virology, University Hospital Essen, Essen, Germany; and; 13 Department of Nuclear Medicine, University Hospital Essen, Essen, Germany, and Department of Molecular and Medical Pharmacology, David Geffen School of Medicine at UCLA, Los Angeles, California

The novel coronavirus disease 2019 (COVID-19) is now considered a pandemic imposing a tremendous challenge on many nuclear medicine departments. Whereas preliminary precautions have been addressed regarding the risk of infection (1), the potential impact of COVID-19 on the risk–benefit ratio of nuclear medicine treatments remains unknown. Here, we discuss risk factors for COVID-19 severity with regard to peptide receptor radionuclide therapy (PRRT), focusing on the question of whether lymphopenia increases risk of infection-related morbidity.

PRRT-associated lymphopenia is a well-established side effect. Grade 3–4 lymphopenia occurs in 75% of patients treated with ^90^Y-DOTATOC (2) and in 18%–52% of patients treated with ^177^Lu-DOTATATE (3,4), with an early nadir 15 d after therapy and subsequent slow partial recovery. Toxicity seems greater after ^90^Y-peptides and appears to be cumulative (5).

The sole study evaluating the impact of PRRT on lymphocyte subpopulations (5) indicated that B cells are predominantly depleted (median reduction, 67%): worse with ^90^Y-DOTATOC (97%) than with ^177^Lu-DOTATATE (49%). The prolonged decrease in B lymphocytes improved in the intercycle period and exhibited partial recovery 3 mo after the last cycle. T lymphocytes were less affected (median reduction, 31%), and natural killer cells only marginally decreased (minimally below lower limits in 2 patients). This explains the lack of severe T- or B-cell–related diseases or other opportunistic infections after PRRT. Other innate immunity cells, for example, macrophages, were not assessed.

The phosphorylated histone variant H2AX (γ-H2AX) is a molecular marker of DNA double-strand breaks, used to estimate the biologic dose of irradiation. Post-PRRT lymphopenia seems to correlate with the blood or bone marrow dose, as documented by the increase in γ-H2AX foci in lymphocytes in all treated patients. The peak number of foci correlates with the absorbed dose to tumor and bone marrow and the extent of peripheral blood lymphocyte reduction (6).

Data emerging from affected countries indicate that severe forms of COVID-19 are associated with profound laboratory alterations, including lymphopenia, thrombocytopenia, and elevated D-dimer, IL-2R, IL-6, IL-10, and tumor necrosis factor α (7,8).

The first study to characterize peripheral lymphocyte subpopulations affected by severe acute respiratory syndrome coronavirus 2 demonstrated that all subtypes were involved (CD4-T, CD8-T, B, and natural killer cells), but a subsequent study showed that CD8-T-cell numbers were primarily reduced (9). Recovery was associated with an increase in CD8-T and B cells (10).

Since lymphopenia is associated with a poor prognosis in the setting of COVID-19, use of procedures with the potential for further immunologic compromise and additional lymphopenia, such as extracorporeal membrane oxygenation, sometimes used during respiratory failure, has been regarded with caution (11).

At this time, it is not known if the moderately compromised immune response (predominantly involving B lymphocytes) associated with PRRT results in an impaired capacity to defend against subsequent severe acute respiratory syndrome coronavirus 2. Despite the demonstrated neutralizing potential of plasma antibodies (12), initial evidence in the development of severe COVID-19 seems to point to a more crucial role for T cells (CD8 and CD4), which are the predominant cells eliminating virus from infected tissue during COVID-19, although they might be involved in organ damage in the later phases of severe infection (13).

Consequently, there is no clear theoretic indication that PRRT places patients at significantly greater risk of acquiring COVID-19 or developing more severe infection-related complications. Anecdotal experiences among us suggest that PRRT-treated patients are not overly represented among our COVID-19–positive patients.

The potential risks of PRRT in patients with progressive neuroendocrine tumor during the COVID-19 pandemic need to be considered in the context of the relative risks and benefits of other available therapies. For example, everolimus is immunosuppressive and may increase the risk of infections, including with opportunistic pathogens. The incidence of infections of neuroendocrine tumor patients treated with everolimus is approximately 20%–29% for all grades and 5%–10% for grades 3 and 4 (14). Similarly, patients treated with temozolomide are at risk for lymphopenia, although risk of opportunistic infections appears to be significant only with dose-dense regimens or with corticosteroids (15,16).

Given the slow and low-dose radiation delivery over time, as opposed to high-dose external-beam radiotherapy or chemotherapy, it is hypothesized that PRRT would have no significant impact on the other hallmark of COVID-19, the coagulopathy related to generalized vasculitis, immune thrombocytopenia, and disseminated intravascular coagulation (17,18).

The kidney is another COVID-19 target, possibly through the angiotensin-converting enzyme receptor 2. Subclinical kidney injury is thought to occur in many COVID-19 patients, severely in about 3% of older subjects with hypertension (19). It is unclear whether the generally subclinical nephrotoxicity produced by prior ^177^Lu-DOTATATE could constitute an additional risk factor in COVID-19–induced renal injury.

Given the evolving nature of this pandemic and the scarcity of data on the subject, the nuclear medicine community is encouraged to prospectively collect and report information on toxicity in patients undergoing PRRT, either before or after COVID-19. At this time, however, routinely monitoring lymphocyte subpopulations would have only research value. Considering the expected rarity of the association of PRRT and COVID-19, we propose a registry under the aegis of the dedicated scientific societies to collect such data and specifically evaluate the potential association between radiation-induced toxicity (hematologic, renal) and COVID-19.

Although available data are scarce, we agree that, for now, PRRT-related lymphopenia does not appear to constitute a strong risk factor for acquiring COVID-19 infection or for developing severe complications. We do, however, recommend careful vigilance regarding the incidence and clinical course of COVID-19 cases in patients undergoing PRRT, and we recommend postponing treatment in active COVID-19 infection. Continuous consideration should be given to the risk–benefit ratio of PRRT during this pandemic, accounting for the geographic prevalence of COVID-19 in the patient’s area as well as patient frailty and comorbidities that may impact pulmonary and renal complications. A few weeks’ delay in highly affected areas for individuals with slowly progressing tumors or with severe comorbidities can be considered, whereas patients with aggressive tumors or those living in scarcely affected areas should receive treatment with no delay.

## DISCLOSURE

Lisa Bodei has an unpaid position on a consultancy or advisory board for AAA, Ipsen, Curium, and Clovis Oncology and performs research for AAA. Emily Bergsland has an unpaid position on an advisory board for AAA. Wouter de Herder is on an advisory board for AAA-Novartis and Wren labs; receives speaker fees from Ipsen, Pfizer, AAA-Novartis, and Novartis; and is on a steering committee for NETTER 2. Diego Ferone is on a steering committee for NETTER 2. Rodney Hicks holds stocks in and is a scientific advisor to Telix Pharmaceuticals (all proceeds donated to his institution). Thomas Hope is on a consultancy or advisory board for Curium and Ipsen; performs research for Clovis Oncology and Philips; and is a trial participant for Novartis and AAA. Marianne Pavel receives honoraria for presentations and a consultancy from Novartis, IPSEN, AAA, Pfizer, and Lexicon. Diane Reidy-Lagunes is on an advisory board for AAA and Lexicon and performs research for Novartis, Ipsen, and Merck. Jens Siveke receives research funding from Celgene and BMS; is on a consulting or advisory board for Celgene, AstraZeneca, and Roche; receives travel funds from Roche, Celgene, BMS, AstraZeneca, and Servier; and holds ownership in FAPI Holding (<3%). Jonathan Strosberg is on a speaker’s bureau for Lexicon and Ipsen and is a consultant for Novartis. Ken Herrmann is a consultant for Bayer, Sofie Biosciences, SIRTEX, AAA, Curium, Endocyte, BTG, Ipsen, Siemens Healthcare, GE Healthcare, Amgen, Novartis, and Ymabs and holds stock options (<1%) in Sofie Biosciences. No other potential conflict of interest relevant to this article was reported.
